# A Rare Entity of Benign Recurring Mesenchymal Tumor of the Female Urethra

**DOI:** 10.1155/2013/698456

**Published:** 2013-09-18

**Authors:** Onur Telli, Haşmet Sarıcı, Berat Cem Özgür, Cem Nedim Yücetürk, Mehmet Ali Karagöz

**Affiliations:** Ankara Training and Research Hospital, Ulucanlar Cadde No. 89 Altındag, Ankara, Turkey

## Abstract

This is a case report of a 51-year-old female patient with benign mesenchymal tumors of paraurethral region which caused lower urinary tract symptoms. The pathological examination of the lesion was reported as angiomyxoma which is a distinct soft tissue tumor characterized by the presence of prominent myxoid matrix and numerous thin-walled blood vessels. This tumor has a predilection for the trunk, head and neck, extremities, and genitalia. It is a benign tumor, and total excision is curative. Recurrence is rare except for aggressive angiomyxomas.

## 1. Introduction

Benign mesenchymal tumors and tumor-like lesions of urethra are rare tumors and mostly arise from the soft tissue of the pelvic region in adult females, and various mesenchymal lesions may affect the because of their apparently shared origin from mesenchyme. We report a rare recurring mesenchymal neoplasia and represent a case by which it comes lower urinary tract symptoms because of an obstruction.

## 2. Presentation of Case

**Figure 1:**
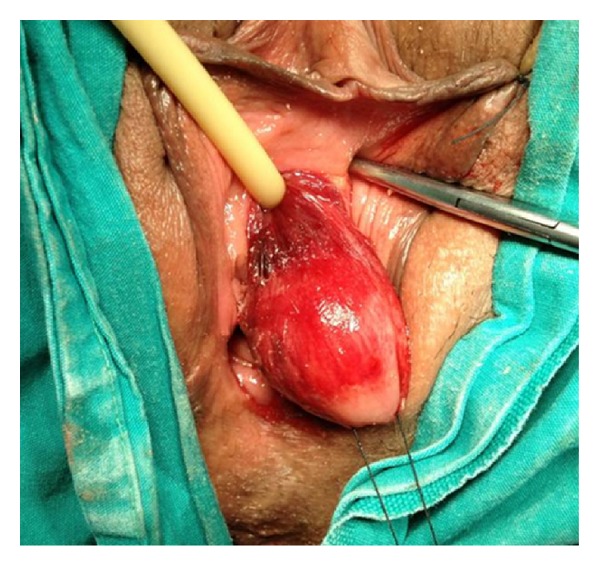
Paraurethral mass through vulva.

**Figure 2:**
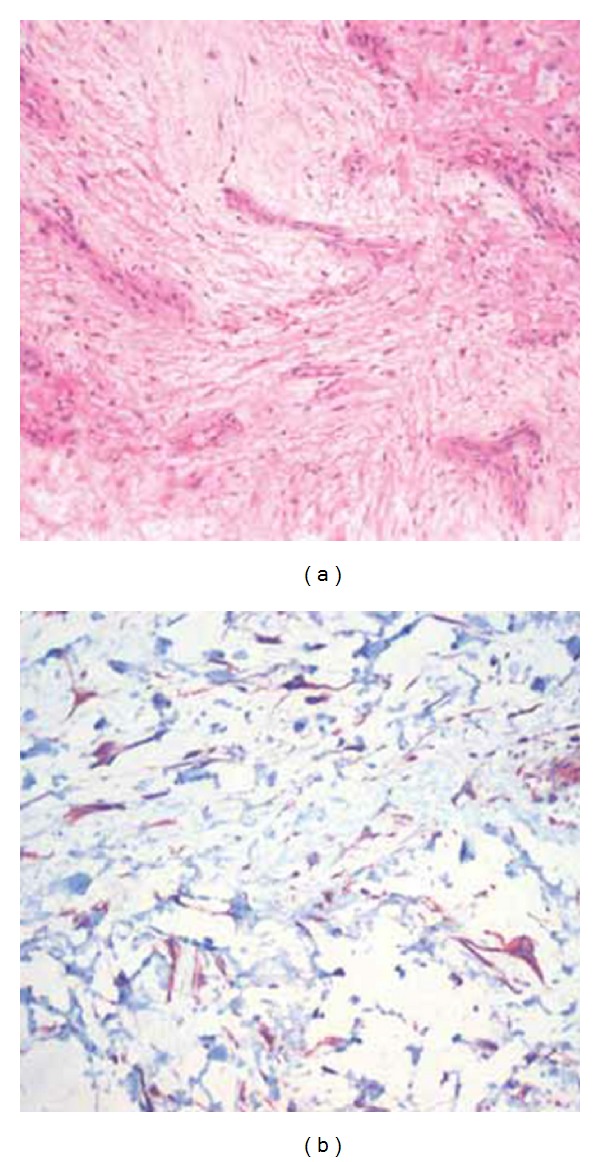
(a) Tumour with multiple thin-walled blood vessels and rarely stellate cells (hematoxylin and eosin, ×40). (b) Spindle-shaped vimentin positive tumour cells (×400).

We present case of a 51-year-old woman who admitted to our hospital with painless mass and progressively increasing voiding difficulties that involve initiating micturition, incomplete bladder emptying, intermittent stream, and massive hematuria. On physical examination, we observed a palpable mass near the urethral opening (Figure 1). Magnetic resonance imaging (MRI) of the pelvis showed the 3.5 × 3.0 cm para-urethral tumor. The tumor was excised, and cystoscopy was performed. Cystoscopy revealed a normal urethra and bladder mucosa. The pathological examination of the lesion revealed benign mesenchymal tumor and was diagnosed histopathologically as angiomyxoma (Figure 2). Histology and immunohistochemistry diagnosis confirms that of a benign lesion. The immunohistochemical profile was intensely positive for vimentin, desmin, CD34, and bcl-2 and entirely negative for CD117 (c-kit), S-100, Pan CK, Kaldesmoni cd31, actin, and cd68. Ki 67 proliferation index was lower than %1. The patient showed a 1.5 × 1.0 cm paraurethral mass again 3 weeks after the first resection, and tumor was resected. 

## 3. Discussion 

Superficial angiomyxoma is a rare mesenchymal benign tumor arising from soft tissue of the pelvis and perineum. It is not thought of as cancer because it usually grows quite slowly and does not usually spread to other parts of the body. But there is an aggressive type that grows more quickly and involves the genital, perineal and pelvic regions of women, with great incidence occurring in the fourth decade [1]. Among the 28 cases of superficial angiomyxoma was first described by Allen et al., estrogen receptor status mostly negative and recurrence is seen within epithelial component positive as reported pathologic examination of our case [2–4]. The diagnosis of angiomyxoma is usually made by the pathologist and usually difficult to differ histopathologically. In our case, although the mass recurred and recurrence is rare in superficial angiomyxomas, MRI of the pelvis showed no local infiltration and was pathologically reported as benign mesenchymal tumor with negative estrogen receptor, distinguished from aggressive angiomyxoma in differential diagnosis. Although data is scant, its complete excision is crucial for disease eradication [4].
